# The Use of Deep Learning in RNA Therapeutic Development

**DOI:** 10.1021/acsnano.6c02090

**Published:** 2026-06-10

**Authors:** Deepak A. Subramanian, Sophia L. Yao, Alvin Chan, Jacob Witten, Daniel Reker, Daniel G. Anderson, Robert Langer, Giovanni Traverso

**Affiliations:** † Department of Chemical Engineering, 2167 Massachusetts Institute of Technology, Cambridge, Massachusetts 02139, United States; ‡ David H. Koch Institute for Integrative Cancer Research, Massachusetts Institute of Technology, Cambridge, Massachusetts 02139, United States; § Broad Institute of MIT and Harvard, Cambridge, Massachusetts 02142, United States; ∥ Department of Electrical Engineering and Computer Science, Massachusetts Institute of Technology, Cambridge, Massachusetts 02139, United States; ⊥ College of Computing and Data Science, 54761 Nanyang Technological University, Singapore 639798, Singapore; # Lee Kong Chian School of Medicine, Nanyang Technological University, Singapore 639798, Singapore; ¶ Centre of AI in Medicine (C-AIM), Nanyang Technological University, Singapore 636921, Singapore; ∇ Department of Biological Engineering, Massachusetts Institute of Technology, Cambridge, Massachusetts 02139, United States; ○ Department of Biomedical Engineering, 3065 Duke University, Durham, North Carolina 27708, United States; ⧫ Duke Cancer Institute, Duke University School of Medicine, Durham, North Carolina 27710, United States; †† Duke Center for Quantitative Biodesign, Duke University, Durham, North Carolina 27710, United States; ‡‡ Harvard and MIT Division of Health Science and Technology, Massachusetts Institute of Technology, Cambridge, Massachusetts 02139, United States; §§ Institute for Medical Engineering and Science, Massachusetts Institute of Technology, Cambridge, Massachusetts 02139, United States; ∥∥ Department of Mechanical Engineering, Massachusetts Institute of Technology, Cambridge, Massachusetts 02139, United States; ⊥⊥ Division of Gastroenterology, Brigham and Women’s Hospital, Harvard Medical School, Boston, Massachusetts 02115, United States; △ Department of Genetics and Genome Sciences, Case Western Reserve University School of Medicine, Cleveland, Ohio 44106, United States

**Keywords:** RNA, computational modeling, deep learning, neural network, structure, gene editing, delivery, high-throughput screening, vocabulary

## Abstract

Ribonucleic acid (RNA)-based therapeutics have emerged as promising methods of disease treatment due to their ability to target the human genome and influence protein production, their versatility, and their relative lack of toxicity compared to other gene therapies. However, the RNA therapeutic design space is extremely large, encompassing multiple variables, including codon identities, secondary structure, and design of specific regions. RNA therapeutic optimization is difficult due to the impracticality of exploring such a vast design space experimentally. To address this limitation, deep learning methods have been employed to optimize RNA therapeutic development. In this review, we examine the application of deep learning models across three key aspects of RNA therapeutic development (RNA structure prediction, CRISPR activity, and RNA delivery), highlighting major contributions in these fields and analyzing how deep learning model architectures could affect model performance. We then discuss challenges associated with using deep learning for RNA therapeutics, such as computational and data limitations. Finally, we offer perspectives on areas for future exploration, such as emerging model architectures and methods of integration with more advanced high-throughput screening techniques. Ultimately, this review provides an overview of how deep learning is used in RNA therapeutic development and how it can evolve in the future.

## Graphical abstract

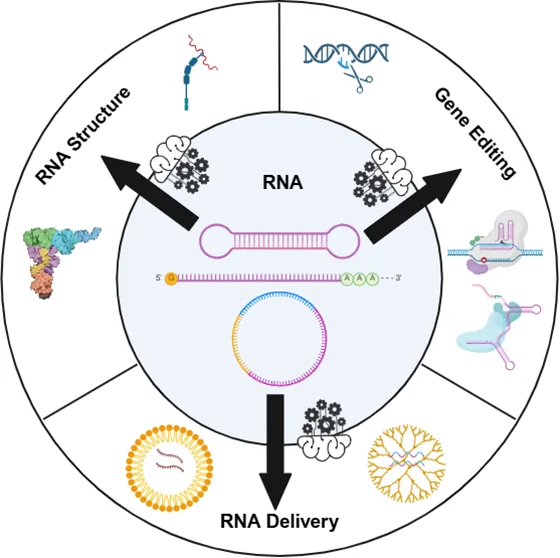

## 1 Introduction

Ribonucleic acid (RNA) is a nucleic acid that serves many functions, such as being an intermediary between cellular DNA (the information repository) and protein production.^1^ Types of RNA in the human body include messenger RNA (mRNA),^2^ transfer RNA (tRNA),^3^ ribosomal RNA (rRNA),^4^ regulatory RNAs, such as micro RNA (miRNA) and small interfering RNA (siRNA),^5^ noncoding and long noncoding RNA (ncRNA) and (lncRNA),^6^ small nuclear RNA (snRNA),^7^ circular RNA (circRNA),^8^ and enhancer RNA (eRNA).^9^ These RNAs serve many essential functions: mRNA facilitates gene expression,^10^ tRNA transfers amino acids to growing polypeptide chains,^11^ rRNA forms the protein-producing ribosomes,^12^ and regulatory RNAs modify transcription and translation in cells.^13^ RNA therapeutics harness or target these cellular RNAs to achieve therapeutic effects.

In general, RNA therapeutics are composed of two core components: (1) payload and (2) vehicle. The “payload” is the RNA sequence carrying the intended therapeutic effect. The “vehicle” encompasses delivery systems that transport RNA payloads to intended cells or tissues, such as polymer-based nanoparticles, viral vectors, or lipid nanoparticles (LNPs), which protect RNA molecules from degradation and facilitate cellular entry, as well as targeting ligands such as GalNAc that are used to direct RNA therapeutics toward their target.

Some RNA therapeutics such as siRNA also have a “target”, which refers to the specific genetic component(s) within cells that the RNA payload aims to regulate, modify, or silence, including disease-causing genes, proteins involved in pathological processes, or specific mRNA sequences requiring modulation. While the “vehicle” can help guide the RNA therapeutic towards its target, most LNPs and GalNAc target the liver, and achieving delivery to other tissues remains a key challenge.

RNA therapeutics offer the ability to target almost any genetic component within cells, many of which are inaccessible to other therapeutic approaches.^14^ While current small molecules and antibodies only target 0.05% of the human genome,^15^ RNA therapeutics can theoretically target any gene of interest. Moreover, once the chemical structure is established and a delivery method determined, RNA-based drugs can be rapidly designed and synthesized for clinical testing. They also demonstrate minimal genotoxic effects compared to DNA therapy due to their non-integrative nature.^16^ The field emerged in the 1990s with antisense oligonucleotides (ASOs) for protein synthesis inhibition, followed by RNA interference (RNAi) and siRNA applications in the 2000s for genome silencing. Recent milestones include FDA approval of the first siRNA therapy Onpattro in 2018, and the 2021 FDA approval of mRNA COVID-19 vaccines. Today, multiple RNA drugs have received approval, with more in clinical trials targeting both rare and common diseases (Table 1). However, challenges persist in predicting engineered RNA activity due to RNA’s structural diversity and environmental sensitivity (to solvents, ions, and metabolites^17^), and insufficient information exists to guide the design of more complex RNA tools.

**Table 1:** List of Some RNA-based Therapies that Have Been FDA-Approved or Are Undergoing Clinical Trials

clinical trial ID	RNA type	delivery mechanism	disease	name, company	FDA approval (Y/N)
NCT05933304 ^18^	mRNA	lipid nanoparticle	COVID-19	Spikevax, Moderna	Y
NCT05815498 ^19^	mRNA	lipid nanoparticle	COVID-19	mNEXSPIKE, Moderna	Y
NCT04368728 ^20^	mRNA	lipid nanoparticle	COVID-19	Comirnaty, Pfizer & BioNTech	Y
NCT05127434 ^21^	mRNA	lipid nanoparticle	RSV	mRESVIA, Moderna	Y
NCT01960348 ^22^	RNAi	lipid nanoparticle	hereditary transthyretin amyloidosis	Onpattro, Alnylam	Y
NCT03338816 ^23^	RNAi	GalNAc conjugate	acute hepatic porphyria	Givlaari, Alnylam	Y
NCT03681184 ^24^	RNAi	GalNAc conjugate	hyperoxaluria	Oxlumo, Alnylam	Y
NCT03400800 ^25^	RNAi	GalNAc conjugate	atherosclerotic cardiovascular disease	Leqvio, Novartis	Y
NCT02292537 ^26^	ASO	naked delivery in solution	spinal muscular atrophy	Spinraza, Biogen	Y
NCT05933577 ^27^	mRNA	lipid nanoparticle	resected high-risk melanoma	V940, Merck & Moderna	N
NCT03417102 ^28^	RNAi	GalNAc conjugate	hemophilia A/B	Fitusiran, Genzyme	N
NCT03847909 ^29^	RNAi	GalNAc conjugate	hyperoxaluria	DCR-PHXC, Novo Nordisk	N
NCT04819269 ^30^	RNAi	eye drops	Sjogren’s syndrome	Tivanisiran, Sylentis	N
NCT04720534 ^31^	RNAi	GalNAc conjugate	hypertriglyceridemia	Plozasiran, Arrowhead	N
NCT05581303 ^32^	RNAi	GalNAc conjugate	cardiovascular disease	Olpasiran, Amgen	N
NCT05677971 ^33^	RNAi	GalNAc conjugate	alpha-1 antitrypsin deficiency liver disease	Fazirsiran, Takeda	N

Deep learning models have drastically transformed many industries, including biology. One particularly notable application is protein structure prediction, where models such as AlphaFold and RoseTTAFold demonstrate exceptional protein structure prediction ability;^10^ the 2024 Nobel Prize in Chemistry was given for this advance. Beyond this application, deep learning approaches have recently shown impressive abilities within RNA biology that promise to accelerate the development of RNA therapeutics.

In this review, we examine advances in deep learning approaches for RNA therapeutics. The main sections analyze deep learning models across key areas in RNA therapeutics, including RNA structural prediction, CRISPR guide RNA on/off target prediction, and RNA delivery technologies. For each application, we examine commonalities in approaches and evaluate individual models’ architectures and performance metrics. Finally, we discuss the challenges and limitations of applying deep learning to RNA therapeutics while exploring emerging trends and future perspectives in this field. While several excellent reviews have addressed deep learning applications in RNA biology,^34,35^ our review uniquely focuses on deep learning applications across all aspects of RNA therapeutics, including carrier design, target selection, and RNA payload design.

## 2 Fundamentals of Deep Learning

Deep learning is a subset of machine learning. Machine learning involves developing algorithms and models that identify patterns in data to make inferences and predictions about unseen data. The process typically consists of training the model on available data to “learn” key dataset features and make predictions for new instances. In deep learning, multiple layers of linear or non-linear modules are “stacked” such that representations from lower architectural levels feed into higher levels (a neural network architecture), enabling more accurate modeling of complex functions and patterns. At a basic level, deep learning architectures take an input embedding (usually as a vector representation, graph representation, or sequence representation), process it via some intricate nonlinear function, and output a different vector, with the shape and meaning of input and output vectors depending on the application in question.

The major deep learning model architectures used in RNA therapeutic development are:

- Recurrent neural networks (RNNs)—neural networks that maintain an internal hidden state, which is updated each time it reads the next character in a sequence to capture sequence structure dependencies. These are primarily used in RNA sequence prediction.
- Convolutional neural networks (CNNs)—neural networks that recognize spatial patterns within data using convolutional layers that learn hierarchical features from data. These are primarily used for image analysis.
- Graph neural networks (GNNs)—neural networks that analyze graph-based inputs. These are primarily used for molecular analysis due to their ability to represent the relationships between neighboring atoms using atoms as nodes and bonds as edges in a graph. In particular, they are able to represent interaction networks between different nucleotides in the RNA sequence, as well as RNA secondary and tertiary structure, due to this ability to construct RNA as a graph. They are also used for molecular deep learning, in areas such as delivery optimization and nucleotide modification.
- Transformers—a relatively new neural network-based architecture which use an “attention” mechanism to identify the most relevant data segments in a sequence (either self-attention, which identifies dependencies between tokens within the same input, or cross-attention, which identifies dependencies between different inputs within a larger feature space). These are primarily used for sequence-based processing, and their inclusion within large language models makes them useful for natural language processing.

While deep learning has proven exceptionally powerful as a prediction tool, the reasons for its predictions are difficult to identify; unlike simpler techniques such as decision trees^36^ and linear regression,^37^ deep learning models are traditionally understood as “black boxes.” However, recent efforts on improving model explainability can provide some insight into the reasoning behind predictions.^38^ These include sensitivity analysis,^39^ integrated gradients,^40^ and Shapley additive explanations,^41^ all aimed at elucidating model learning processes and enhancing interpretability.

Like all machine learning techniques, deep learning is heavily dependent on the quantity and quality of the data. Data processing ensures proper input formatting for deep learning models.

When data availability is limited, as often occurs in RNA therapeutic experiments, data augmentation can sometimes be applied to improve model robustness. Data augmentation artificially expands dataset size by generating additional training data through small modifications to existing data.^42^ Other challenges in data handling include learning from “noisy” data (where the data have large variation in the predicted value) and integrating data from different experimental assay systems.

## 3 RNA Structure Prediction

Although RNA has gained prominence in therapeutics, its structural complexity remains incompletely understood.^43^ Understanding RNA structure is crucial for designing effective RNA-based therapies, as variations in RNA’s primary, secondary, and tertiary structures can significantly impact translation.^44^ Furthermore, additional RNA elements, including non-coding RNA (ncRNA), RNA-binding proteins (RBPs), and untranslated regions (UTRs), play critical roles in therapeutic efficacy. In the context of therapeutic design and development, RNA structure prediction falls under the “Payload” consideration (as structural understanding can inform environmental interactions and translation efficiency^45^). This section examines how deep learning approaches contribute to predicting and understanding these structural aspects of RNA therapeutics.

### 3.1 Secondary and Tertiary Structure

RNA molecules fold into secondary structure based (for the most part) on Watson-Crick base pairing (although some RNA sequences are not based on Watson-Crick base pairing and may not be paired at all) and then further into tertiary structure through interactions among secondary structural elements. Traditionally, researchers required months or years to solve complex RNA structures using experimental methods such as NMR,^46^ X-ray crystallography,^47^ and cryo-electron microscopy.^48^ These conventional prediction methods face limitations due to molecule size and have very high operating costs.^49^ While ab initio techniques attempt to build 3D conformations by simulating RNA folding from scratch, they are constrained by complicated topologies and vast sampling spaces.^50^

Deep learning offers a faster, more cost-effective approach to RNA structure prediction using existing data. Similar success has been demonstrated in protein structure prediction, where AlphaFold^51^ and its successors (AlphaFold2 and AlphaFold3) have predicted over a million structure models, far exceeding historically determined experimental structures. This high-resolution structural information is crucial for developing a comprehensive understanding of RNA structure and folding, ultimately advancing RNA-targeted drug development. However, there is generally a lack of available high-quality data on RNA structures compared to that of proteins, providing a major bottleneck for training RNA structure prediction models (which will be discussed later in the review).

Different types of deep learning architectures have been employed for RNA structure prediction. Transformer architectures are widely used in the latest deep learning models and have become prominent in RNA structural prediction due to their ability to capture long-range interactions.^52,53^ GNNs represent RNA molecules as a connected graph. Nucleotides are often represented as nodes, and base-pairing interactions are edges, allowing structural constraints like canonical pairing rules to be enforced directly within the model architecture.^54^ Other types of models utilize convolutional or hybrid architectures to process image-based RNA structure inputs.^55,56^ In recent years, approaches involving transfer learning, end-to-end approaches, and foundation models have been used for structure prediction.^57,58^

### 3.2 Non-coding RNA

Non-coding RNAs (ncRNAs), while sharing sequence characteristics with mRNA, serve distinctly different functions.^59^ These molecules play crucial roles in cell differentiation^60^ and genomic imprinting^61^ and are implicated in various human diseases, including cancer^62^ and cardiovascular disease.^63^ Understanding ncRNA types is essential for elucidating disease mechanisms and developing more effective treatments. ncRNAs are categorized into long non-coding RNA (lncRNA) and small ncRNA (sncRNA), with deep learning algorithms finding applications in various aspects, from open reading frame detection to lncRNA identification and classification.^64^

Different deep learning approaches have been used to analyze the ncRNA structure. Transformer-based approaches are generally used for classifying ncRNA (such as identifying misannotated lncRNA) and predicting ncRNA subcellular localization.^65,66^ Convolutional architectures have been used for classifying lncRNA.^67^ Hybrid architectures have been used for modeling ncRNA sequences.^59,68^

### 3.3 RNA-Binding Protein

RNA-binding proteins (RBPs) serve as crucial regulators in cell differentiation,^69^ metabolism,^70^ development,^71^ and health. While traditional experimental methods for RBP predictions (RBP-protein binding affinities and identification of binding sites) are time-consuming and inflexible, deep learning approaches offer more efficient solutions with lower financial and time costs while providing insights into RBP amino acid properties that are difficult to obtain experimentally.^72^

Several models utilize transformer architectures for RBP prediction, usually to predict protein-RNA binding affinity.^73,74^ Other models utilize CNN-based and hybrid approaches,^75,76^ which can allow for single-nucleotide resolution. Recently, combinations of CNNs with long short-term memory (LSTM) architectures have been used to predict RNA-protein binding.^72,77^ In addition, structure prediction models such as AlphaFold3 have been able to predict RNA structures and RNA-protein binding.^78^

### 3.4 mRNA Untranslated Region

In addition to the coding sequence of mRNA, mRNA contains untranslated regions (UTRs) at the 5′ and 3′ ends of the coding sequence.^79^ These regions are key determinants of mRNA stability and translation efficiency; the 5′ UTR plays an important role in ribosome loading,^80^ while the 3′ UTR is a target for the binding of RBPs and miRNA that can cleave the mRNA or block its translation.^81^ For this reason, optimization of UTRs is an important subject for mRNA-based therapeutics. To generate training data, researchers generally use large-scale reporter assays based on protein expression generated from reporter genes with varied UTRs or use sequencing data that reports on mRNA stability or active translation.^82^

CNNs have proven successful for UTR design,^83,84^ possibly because UTRs are relatively short, which attenuates the advantage of transformers. Other models have used transformer-based models to predict translation efficacy from the UTR structure.^85,86^ These and future models are essential for designing precisely targeted, efficiently translated, stable mRNAs for mRNA-based gene therapy.

### 3.5 RNA Modification Pattern Prediction

The RNA structure can be modified during epigenetic modification through the action of various chemicals, such as N^6^-methyladenosine, 5-methylcytosine, 5-methyluridine, and 5-hydroxymethylcytosine, among others. Understanding the effect of these chemicals can help predict where post-translational modifications take place, which can improve our understanding of how RNA sequences are translated into functional proteins^87^ or contribute to disease progression.^88^ Deep learning has been employed to predict the presence of RNA modification sites or the effect of RNA modification on the resulting sequence.

Standard feed-forward neural networks have been used to identify RNA modification sites for specific RNA modifiers such as 5-methyluridine, showing the ability to predict where these modifiers can alter the RNA structure based on sequence information and features.^89^ Other types of neural networks, such as RNNs^90^ and GNNs,^91^ have also been employed. Recently, transformers^92,93^ and combinations of CNNs and transformers^94^ have been employed; these take advantage of the transformer’s capability to accurately utilize whole–sequence interactions for predictions, while the hybrid models also incorporate the immediate interactions between neighboring nucleotides.

### 3.6 Model Architecture Analysis

Transformer-based architectures are rapidly increasing in popularity for RNA structure prediction tasks. Their ability to capture long-range dependencies and model complex relationships between nucleotides through self-attention mechanisms makes them particularly well suited for learning the base-pairing interactions that guide RNA folding.^95^ Additionally, the use of foundation models pre-trained on large, unlabeled RNA datasets has become increasingly popular, enabling accurate downstream predictions, even in data-limited settings.^96^ GNNs are often used when secondary structure information is available and is well suited for tasks like predicting tertiary contacts or modeling loop penalties.^97^ In contrast, CNNs struggle with capturing long-range sequential dependencies due to their fixed receptive fields;^98^ even though stacked convolutions can theoretically mitigate this issue, they can remain susceptible to gradient instability.^99^ RNNs, meanwhile, encounter gradient issues when modeling distant interactions^100^ and have lower training efficiency due to their need for sequential computation.^101^ Consequently, these architectures perform less effectively for tertiary structure prediction and complex RNA folding tasks.

Representative models for structure prediction include trRosettaRNA, which takes RNA sequences and MSAs as input and uses a transformer network to predict inter-nucleotide distances and angles. It achieves competitive performance in CASP15 blind tests and RNA-Puzzles experiments but struggles to predict accurate structures with synthetic RNAs.^50^ NuFold uses an AlphaFold2-inspired transformer architecture to go from input RNA sequence to 3D atomic structure, outperforming state-of-the-art energy-based methods and demonstrating a particular advantage in building correct local geometries of RNA. However, due to its transformer architecture, it struggles on more novel RNA families with limited training examples.^58^

For ncRNA classification, lncRNAnet uses an RNN to model input RNA sequences and a CNN-based ORF indicator to distinguish lncRNAs from protein-coding transcripts. A limitation mentioned in the study is a sensitivity-specificity trade-off, where the small number of annotated lncRNA transcripts leads to low specificity in identifying true lncRNAs.^59^

For RBP prediction, HydRA uses protein sequences as input and combines CNNs, support vector machines (SVMs), and a transformer-based protein language model to predict RNA binding capacity. It demonstrates high specificity and sensitivity across characterized and uncharacterized RBPs and predicts hundreds of novel RNA-binding-associated domains. However, it relies heavily on the density of protein–protein interaction networks to make predictions, so performance degrades when PPI networks are sparse.^75^

For UTR design, UTR-LM uses a transformer-based language model to predict mean ribosome loading, translation efficiency, and mRNA expression level using input 5′ UTR sequences. It outperforms the best-known benchmark for the listed tasks by up to 5% and was also used to design a library of novel 5′ UTRs with a 32.5% increase in protein production relative to established 5′UTRs optimized for therapeutics.^86^

Figure 1 shows a comparison of the transformer and traditional neural network models.

**Figure 1:**
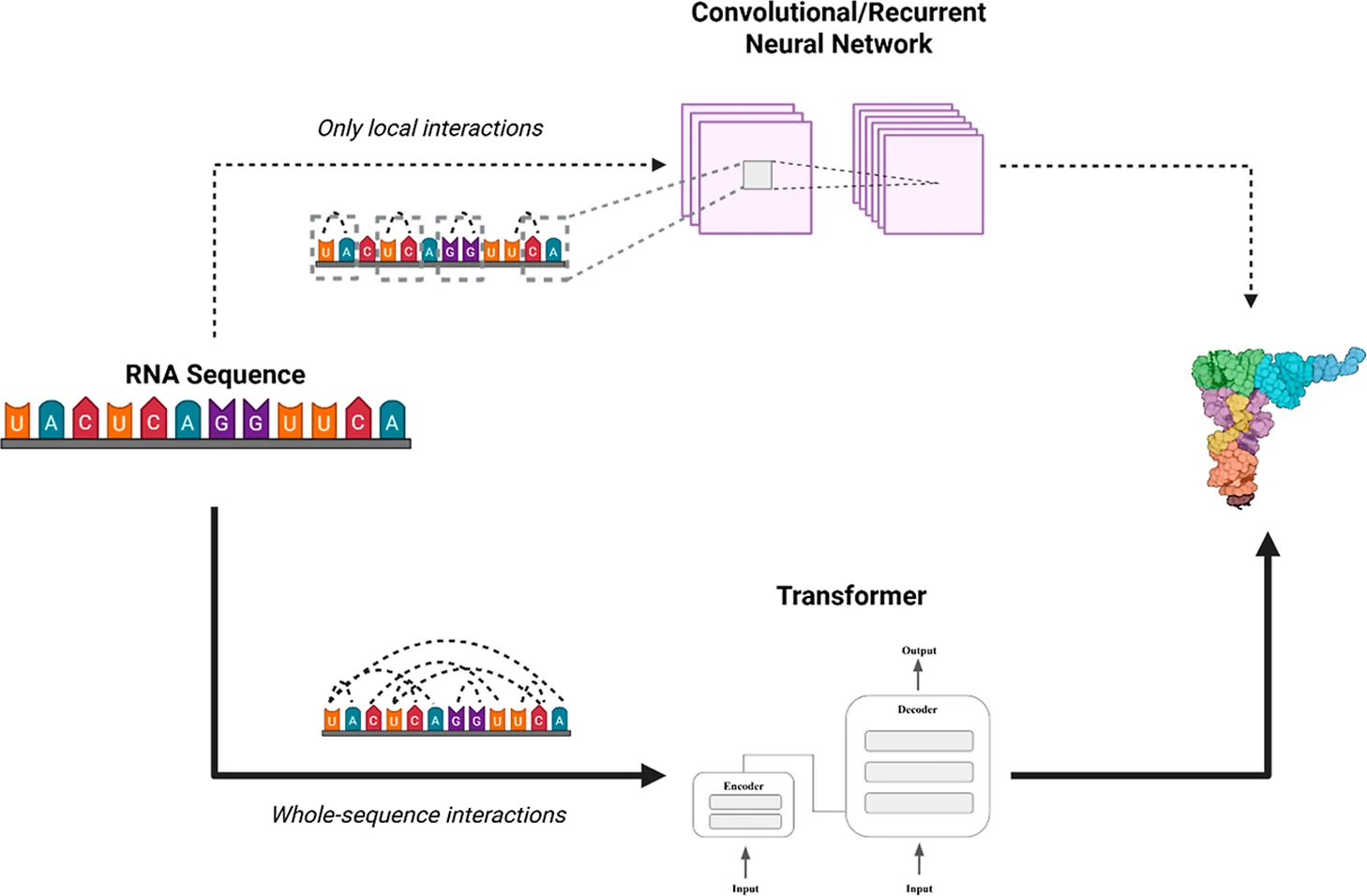
Comparison of the traditional neural network and transformer approaches for RNA structure prediction. While traditional neural networks have typically only considered local interactions between nucleotides, making their predictions weaker, transformers have the capacity to consider whole–sequence interactions based on their use of the self-attention mechanism, making them better suited for RNA structure prediction tasks. Created in BioRender. Traverso, G. (2025) https://BioRender.com/a7okeb5.

## 4 Gene Editing with CRISPR

CRISPR-Cas-based systems have emerged as prominent RNA-based therapeutics for gene editing in the past decade. These systems utilize enzymes that can generate selective breaks in DNA structure, enabling site-specific modifications and gene editing capabilities.^102^ Most notably, techniques called base editing and prime editing allow for targeted genome edits without inducing double-stranded breaks, improving the precision and safety of genome editing. CRISPR systems’ versatility and relative simplicity have led to widespread adoption across biomedical applications.^103^ However, certain aspects of CRISPR-Cas technology remain challenging to predict, and better understanding of these elements could enhance gene editing technology development and implementation. This section examines deep learning applications in two critical aspects of gene editing: predicting CRISPR-Cas system targeting capability and forecasting base editing and prime editing outcomes.

### 4.1 CRISPR On/Off Target Activity Prediction

A critical challenge in CRISPR-Cas9 system development is accurately predicting sgRNA on-target knockout efficiency and off-target profiles. While current detection methods include CIRCLE-seq^104^ and whole-genome sequencing (WGS),^105^ these approaches face limitations due to high sequencing costs and insufficient complexity to account for intranuclear microenvironments.

Some models combine transformer architectures with other approaches for improved predictions,^106,107^ possibly due to their ability to account for short-range and long-range interactions between nucleotides. Other models use CNN-based architectures,^108,109^ possibly due to their ability to represent RNA-DNA interactions as computer images. Recurrent network-based models have also been used in combination with other models such as CNNs,^110,111^ possibly due to the complementary mechanisms of action of CNNs and recurrent networks. Recently, methods that combine CNNs and transformers as well as novel encoding methods have been employed to further improve their on/off-target activity prediction.^112,113^

### 4.2 CRISPR Base Editing and Prime Editing

Base editors (BEs) combine a DNA-targeting CRISPR-Cas module with adenosine or cytosine deaminase, enabling CG to TA base pair transitions and vice versa.^114^ Prime editors (PEs) fuse Cas9 to a reverse transcriptase and use extended guide RNAs called pegRNAs to directly write programmable edits into the genome.^115^ While both techniques show promise as genome editing tools, their application is currently limited by variable editing efficiencies across genomic loci.^116^ Deep learning models have emerged as valuable tools for predicting editing outcomes, supporting both basic research and translational applications.

Recent models leverage advanced architectures for improved predictions. Some models utilize attention-based methods such as transformers and recurrent neural networks to identify the most important BE, PE, and pegRNA components associated with editing efficacy.^117,118^ Other models use various types of neural network architectures such as CNNs and RNNs to predict base editing and prime editing efficiencies and outcomes.^119,120^

### 4.3 CRISPR Design Generation

Deep learning has enabled the generation of novel CRISPR-Cas proteins, as demonstrated by OpenCRISPR-1,^121^ a machine learning-designed Type II effector that assembles as a functional gene editor in human cells, marking the first instance of genome editing by an entirely AI-generated CRISPR system and expanding the potential for data-driven CRISPR engineering. This approach leverages a protein language model pre-trained on diverse CRISPR-Cas sequences, followed by fine-tuning to capture family-specific functional constraints, enabling the design of effectors that diverge significantly in sequence space while retaining genome editing functionality. Also leveraging deep learning for CRISPR design, Evo,^122^ a fine-tuned language model trained on 72,831 CRISPR-Cas loci, generates functional CRISPR-Cas systems by capturing covariation patterns between protein and ncRNA components, enabling the design of novel Cas9 variants and guide RNAs with enhanced activity and structural diversity beyond naturally occurring homologs.

### 4.4 Model Architecture Analysis

CNNs were initially the leading architecture for gRNA prediction and design, driven by their inductive biases toward local pattern recognition and their effectiveness with relatively small datasets. However, hybrid transformer-CNN architectures have recently emerged as state-of-the-art in this space, as they integrate the strengths of both approaches.^123,124^ In these hybrid models, convolutional layers extract local sequence motifs and positional information while transformer components employ self-attention mechanisms to learn extended sequence correlations between the motifs of interest. The increasing availability of large, high-quality datasets generated from high-throughput sequencing and other experimental platforms has further facilitated the adoption of transformer-based models. Figure 2 shows a schematic of a hybrid transformer-CNN architecture for CRISPR design.

**Figure 2:**
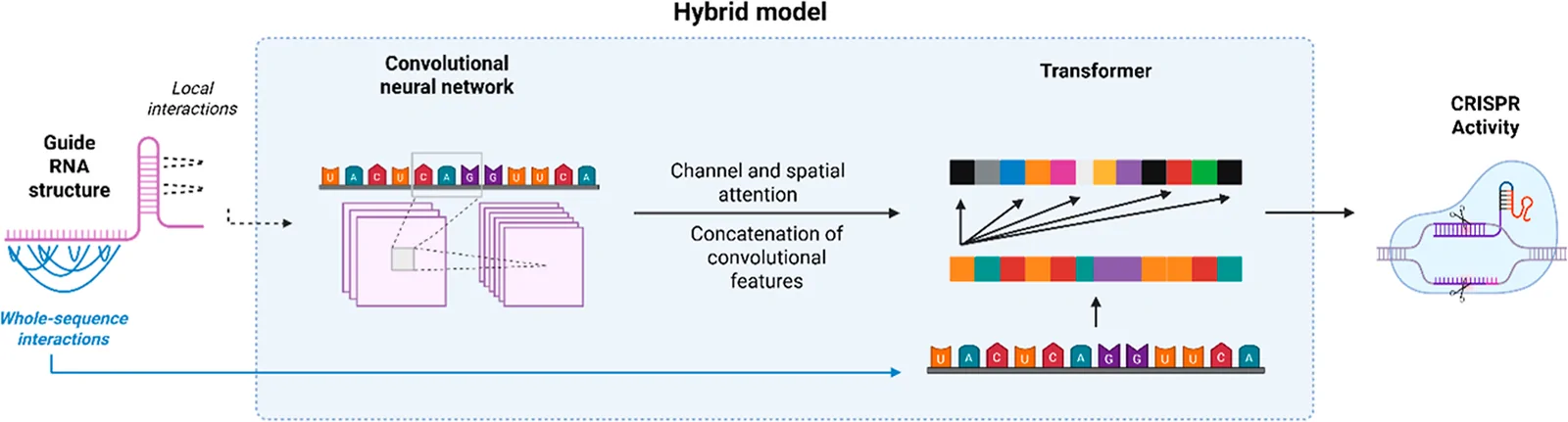
Schematic describing the use of hybrid transformer-CNN model architectures for CRISPR activity prediction based on guide RNA structure. The CNN has primarily been used due to its ability to consider local interactions, but hybrid architectures with transformers have been developed that can also consider whole–sequence interactions. Figure based on architecture from Emtiaj et al.^123^ Created in BioRender. Traverso, G. (2025) https://BioRender.com/jbwt5uj.

Representative models for on/off target prediction include CRISMER, which uses a hybrid CNN-transformer architecture to encode sgRNA-DNA pairs as sparse one-hot matrices, then uses convolutional layers to extract local k-mer features and a transformer block for long-range dependency modeling.^123^ TIGER is an earlier CNN-only model that encodes sgRNA-DNA pairs as image matrices and outputs off-target predictions; however, with only convolutional layers, TIGER is limited in its ability to capture distal sequence contexts.^108^ For base editing prediction, BE-DICT uses an attention-based deep learning algorithm that takes target sequences as input and predicts editing efficiency and position-specific editing outputs for adenine and cytosine base editors. Its strength lies in its versatility, but since it was trained on lentiviral integrated sequences, its predictions struggle to generalize to endogenous genomic loci with different chromatin contexts.^116^ For CRISPR design, Evo uses a long-context language model trained on CRISPR-Cas loci to generate novel Cas9 variants and guide RNAs. It excels in modeling co-evolutionary dependencies across the CRISPR-Cas system but struggles to maintain coherent and diverse generation over long sequences.^122^

Graph-CRISPR is a graph-based model that takes an sgRNA sequence and secondary structure as input and outputs predicted sgRNA editing efficiency. It surpasses baseline models across systems like CRISPR-Cas9, prime editing, and base editing, but its performance depends heavily on the quality of the input secondary structure.^125^

## 5 RNA Delivery

RNA delivery, particularly mRNA delivery, has gained prominence following the development of SARS-CoV-2 mRNA vaccines.^126^ This has accelerated efforts to develop delivery methods for various RNA-based therapeutics, including circular RNA and small interfering RNA.

Lipid nanoparticles (LNPs) have emerged as particularly promising vehicles for clinical mRNA delivery.^127^ Because of their structural similarity to cellular membranes (which allows them to more effectively transfect cellular targets and successfully deliver mRNA), LNPs have been employed to deliver RNA cargoes in a variety of different applications, including delivery to targets such as the liver,^128^ spleen,^129^ lungs,^130^ bone marrow,^131^ and placenta.^132^ However, the expansion of RNA therapies beyond SARS-CoV-2 vaccines has been limited by the lack of cell-type-specific LNPs. In addition, the tissue-specific LNPs have not yet been translated into clinical therapies.

Traditional LNP development relies heavily on trial-and-error approaches. While most optimization efforts have focused on high-throughput combinatorial screening of different modalities,^133^ deep learning offers an alternative (and complementary) approach. These methods enable rapid design, synthesis, and evaluation of ionizable lipids for mRNA delivery from extensive candidate libraries, revealing important properties such as cell-specific preferences and optimal tail lengths that can accelerate customized LNP development.

Recent models employ various architectures for LNP design and optimization. Some models use neural network-based methods^134^ to predict how changing ionizable lipid structures affected transfection efficacy.^135^ Other models use transformers to incorporate molecular and nanoparticle features into the input, using these factors to help predict delivery efficacy.^136^ Transformer-based architectures have recently become widely applied in modeling RNA delivery efficacy, particularly in systems that learn knowledge from whole formulations,^137^ because of their capacity to capture complex interactions within multicomponent systems and to integrate diverse, multimodal data sources.

Representative models for RNA delivery include AGILE, which uses a GNN pretrained on the molecular graphs of virtual lipids and fine-tuned on experimental LNP transfection measurements to predict cell-type-specific delivery preferences. Its main limitation is that it only models the ionizable lipid components, leaving interactions between other LNP components like the helper lipid, sterol, and PEG lipid unaccounted for.^134^ COMET represents entire LNP formulations as tokens in a transformer architecture and identifies candidates with high in vitro and in vivo transfection efficacy. However, its transformer architecture and molecule representations require greater amounts of labeled data and computational resources than models that only consider a single molecular component.^137^

Future improvements might include designing computational architectures that can incorporate other novel materials such as polymers or targeting ligands to make predictions on delivery efficacy, as well as simultaneous exploration of mRNA cargo design^82^ to simultaneously optimize vehicle and payload design for optimal transfection and protein production.

Figure 3 shows the process of applying deep learning models (neural networks and transformers) to design new lipids and lipid nanoparticles for mRNA delivery.

**Figure 3:**
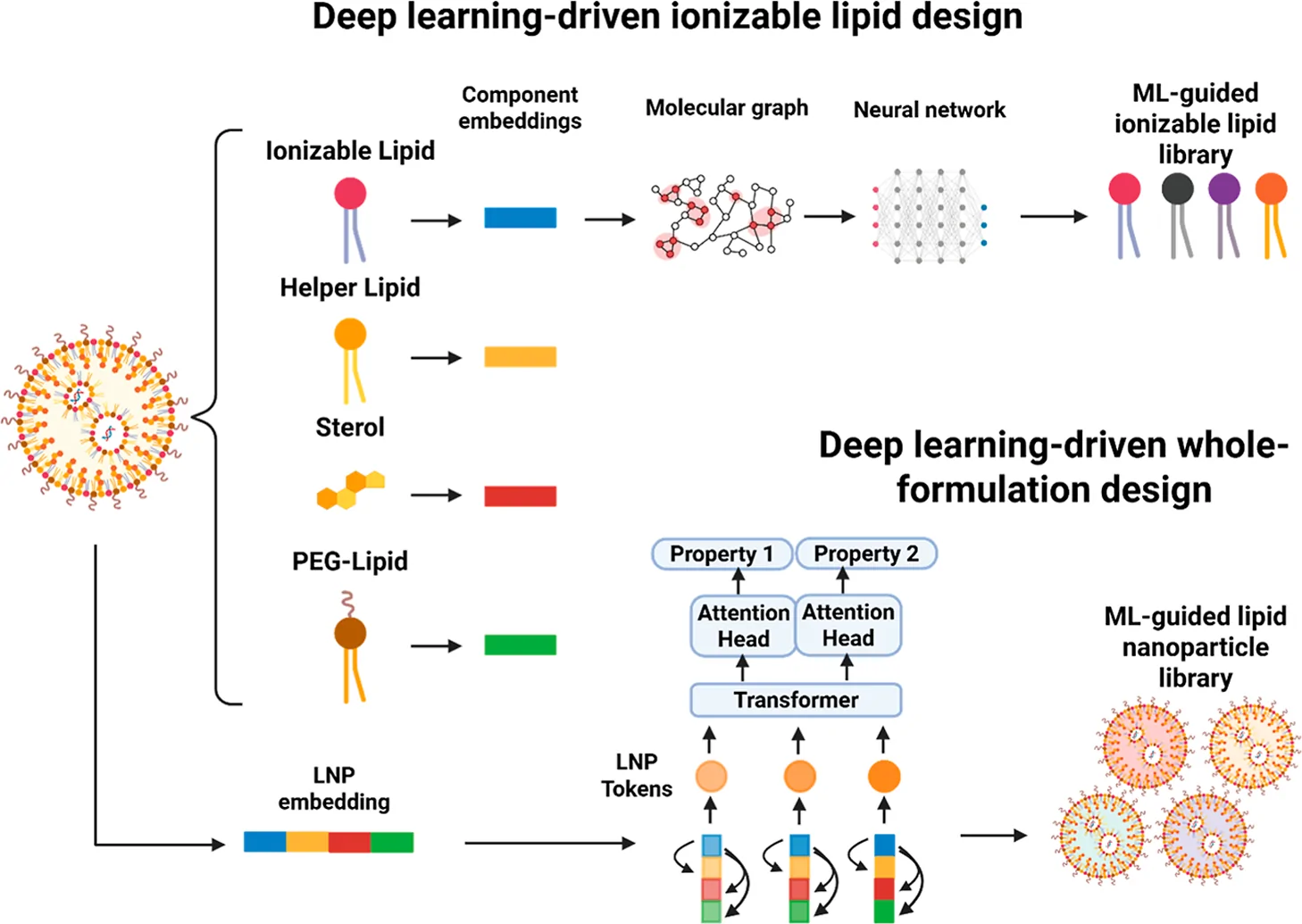
Use of deep learning models to design new components for mRNA delivery. Lipid nanoparticles are composed of multiple components. Neural network-based methods have been developed to design novel ionizable lipids (one of the components) with greater transfection efficacy. Other methods use transformers to model the entire nanoparticle, leading to the design of novel formulations with higher efficacy. Created in BioRender. Traverso, G. (2025) https://BioRender.com/250uzz9.

Table 2 lists the representative models for the different RNA therapeutic applications shown in this review.

**Table 2:** Representative Models for Different RNA Therapeutic Design Applications

application domain	model name	architecture	input features	output task	performance metric
RNA 3D structure	trRosettaRNA^50^	transformer	sequence, multiple sequence alignment (MSA)	3D coordinates (distances, angles)	outperforms other deep learning based methods in CASP15 when measured by RMSD
RNA 3D structure	NuFold^58^	transformer	sequence, MSA, 2D structure	All-atom 3D coordinates	full-atom RMSD of 5 Å or less on RNA structure prediction tasks
ncRNA classification	lncRNAnet^59^	RNN + CNN	sequence, open reading frame indicator	lncRNA vs. coding RNA classification	outperforms the best alternatives by 7.83% (specificity), 5.76% (accuracy), 5.30% (F1-score), 3.78% (AUC)
RBP prediction	HydRA^75^	CNN + SVM + transformer	protein sequence, interaction graph	RNA-binding capacity	AUROC = 0.866, AUPRC = 0.682
UTR design	UTR-LM^86^	transformer	5′ UTR sequence	translation efficiency	outperformed best-known benchmark by 8% for translation efficiency and mRNA expression level prediction
CRISPR on/off target	CRISMER^123^	hybrid CNN-transformer	sgRNA-DNA one-hot matrix	off-target cleavage score	F1 = 0.7092, PR-AUC = 0.8006
CRISPR on/off target	TIGER^108^	CNN	one-hot encoded guide, target sequence, flanking context	on/off target activity score	AUC up to 0.95
CRISPR base editing	BE-DICT^116^	attention + RNN	target sequence	editing efficiency, editing outcome	Pearson *R* ^2^ = 0.92 (ABE8e)
CRISPR Design	Evo^122^	long context language model	CRISPR-Cas loci sequences	novel Cas9 variants, guide RNAs	generative model, evaluated by functional validation
CRISPR design	Graph-CRISPR^125^	GNN (GIN)	sgRNA sequence, secondary structure graph	editing efficiency (Cas9, BE, PE)	Pearson *R* ^2^ = 0.78 for prime editing and 0.94 for base editing
RNA delivery	AGILE^134^	GNN (GIN)	molecular graph (ionizable lipid)	transfection efficiency	Pearson *R* ^2^ = 0.78 for mFluc expression prediction in HeLa cells
RNA delivery	COMET^137^	transformer	full LNP formulation (all components, ratios)	transfection efficacy	Spearman *R* = 0.873, Pearson *R* = 0.866

## 6 Challenges and Limitations

Previous sections have demonstrated how deep learning methods have transformed various aspects of RNA therapeutic design and development. While these advances are significant, understanding and addressing the limitations of deep learning approaches is crucial for their effective implementation in this field, and integrating emerging trends can address some of these limitations.

### 6.1 Computational Limitations

#### 6.1.1 Resource Requirements and Computational Costs

Deep learning models, particularly large models with correspondingly large datasets such as protein or DNA language models,^122,138^ require substantial computational resources due to their large number of trainable parameters. While simpler machine learning approaches such as linear regression,^139^ random forest,^140,141^ and SVMs^142,143^ can operate effectively on central processing units (CPUs), deep learning methods typically are trained much more quickly on graphical processing units (GPUs) and tensor processing units (TPUs). Although efforts have been made to reduce computational demands through parallel processing across multiple GPUs and TPUs, the financial implications of these hardware requirements can create significant barriers to model development and implementation in some cases.

#### 6.1.2 Model Complexity and Data Requirements

Model complexity presents two major challenges:

1. Data requirements: Deep learning models require extensive training data due to their numerous parameters and have an essentially bottomless appetite for data due to recent models’ ability to scale. While advances have been made in developing more data-efficient models,^144^ the field still requires further improvements to reduce data dependencies, particularly for applications with limited data available.
2. Computational efficiency: The increased size of deep learning models leads to greater computational costs and longer training times.^145^ These factors can limit the practical application of deep learning approaches in therapeutic design.

As the field shifts towards a more diverse range of model architectures, these data and computational limitations become more relevant. Transformer models require larger training datasets than CNNs to avoid overfitting, so it is currently difficult to apply transformers effectively for tasks in data-limited domains like base editing or RNA delivery.^145^ GNNs are more data-efficient than transformers but require high-quality input graphs to achieve greater performance.^146^ Balancing these considerations will require the continued development of hybrid architectures and training paradigms like transfer learning and semi-supervised learning.^147,148,149^

### 6.2 Data Limitations

Training data is essential for deep learning approaches in RNA therapeutics and can be generated through a range of different approaches. Ground truth RNA structure prediction data is derived from techniques like NMR, X-ray crystallography, and cryo-EM.^46,47,48^ Data for UTR-related tasks often come from large-scale reporter assays tracking protein output or mRNA stability in cells expressing reporter genes driven by specific UTR sequences.^84^ CRISPR editing data are generated by high-throughput sequencing of cells treated with guide RNA libraries.^116^ LNP delivery models use high throughput transfection screens where libraries of LNP formulations encapsulating reporter mRNAs are synthesized and assayed in cell lines or in vitro.^134,136^

#### 6.2.1 Available Data Resources

Several existing databases provide publicly accessible data for RNA therapeutic research:

1. RCSB protein data bank: Manages 3D structural data for proteins, nucleic acids, and complex biomolecules.^150^ This provides structural information that can be used as ground-truth labels for training models to predict the RNA structure from its nucleotide sequence.
2. AURA: Houses information on over 100,000 RNA sequences, 1000 regulatory factors, and 2.5 million binding sites, including circular RNA regulatory data.^151^ This provides sequence data that can be used to train models that can design new RNA sequences, including UTRs, which provide optimal translation efficacy.
3. Nucleic acid database (NDB): Contains experimental data from over 100 RNAs, 2500 protein-RNA complexes, and 185 RNA-ligand complexes, with RNA structure analysis tools.^152^ This can be used to design RNA-binding proteins and understand their interactions, and it provides experimentally derived structural information that can be used as ground-truth labels for training RNA structure prediction models.
4. AANT: Collection of over 10,000 amino acid and RNA interactions, including datasets on post-translational regulation, thermodynamics, binding pairs, and 3D protein-nucleic acid interactions.^153^ This can be used to train models that can identify and design RNA-binding proteins.
5. RNAMDB: Collection containing detailed data on the 109 known post-transcriptional RNA modifications, with information about the chemical structure of the modified nucleoside, common name/symbol, searchable Chemical Abstracts registry numbers and index name, elemental composition, molecular weight, phylogenetic source of the RNA, and type of RNA.^154^

#### 6.2.2 Data Limitations

Despite these resources, RNA therapeutic development faces significant data limitations. While databases such as RNA STRAND exist for RNA structural data,^155^ the scarcity of high-quality annotated data (particularly in newer areas such as RNA delivery and gene editing) poses a primary challenge, as obtaining extensive labeled datasets remains difficult due to domain complexity and novelty.^34^ This limitation impacts model learning capacity and reduces generalizability. As such, additional experimental data is usually necessary to ensure that the model is trained on a representative data set and can generalize well to novel sequences or delivery vehicles. While there can be a tradeoff between additional experimental data and model predictions if the additional data confuses the model (which can particularly be an issue when combining data from multiple sources due to differences in experimental conditions), adding additional data generally improves model performance.

In addition to training data, experimental validation is necessary to have confidence in model predictions. This involves performing the same (or similar) experiments on candidates identified from in silico screening or novel RNA therapeutic generation, which requires additional experimental capabilities; this makes it challenging for researchers to have full confidence in the outputs of their models.

A significant challenge lies in the disparity between in vitro and in vivo training data, as well as between training data and real-world clinical data.^156^ Models trained on data obtained from a specific tissue or species generally have trouble transferring their insight across tissue types or species. Models trained on controlled experimental data often struggle to generalize to clinical environments with greater variability.^157^ Additionally, dataset biases, including demographic factors like race and sexual orientation, can limit model universality.^158^

These challenges necessitate:

1. Development of more representative datasets.
2. Methods to mitigate inherent biases.
3. Improved benchmarking procedures, particularly in gene editing and RNA delivery.
4. Better alignment between experimental and clinical data.

Ultimately, identifying gaps in data quality and developing methods to collect high-quality data is necessary to improve the performance of deep learning models, particularly data-hungry models such as transformers and complex neural networks.

## 7 Outlook and Future Trends

### 7.1 Integration with Other Technologies

The integration of deep learning with other technologies has facilitated the creation of sufficient data to improve deep learning model performance. For example, recent advances in sequencing technologies have significantly enhanced deep learning model accuracy. Single-cell RNA sequencing (scRNA-seq) and spatial transcriptomics provide high-resolution gene expression profiles and spatial context, enabling better understanding of cellular states and microenvironments.^159^ Long-read sequencing generates comprehensive RNA sequence data, including isoforms and structural variants, improving structure–function relationship predictions.^160^ High-throughput screening enables rapid evaluation of biological activity across vast numbers of genetic sequences, generating extensive training data for model development.^161^

In addition, the development of advanced chemical and delivery techniques further supports therapeutic development. Combinatorial chemistry enables the creation of extensive libraries of modified nucleotides and small molecules, enhancing RNA therapeutic stability, delivery, and efficacy.^162^ High-throughput LNP synthesis facilitates rapid screening of diverse formulations, working synergistically with deep learning models to optimize the composition and size of delivery vehicles.^163^

Finally, the combination of multiple advanced techniques can enhance model capabilities. For example, CRISPR-based sequencing provides high-confidence gene editing outcome data, improving therapeutic targeting predictions for base and prime editing based on editor efficiency and off-target effects.^164^

### 7.2 Large Language Models (LLMs) and Their Uses in RNA Therapeutic Design

In particular, large language models (LLMs), such as Tx-LLM,^165^ have the potential to transform how we view medicine and research by providing unprecedented capabilities in data analysis, hypothesis generation, and knowledge synthesis. These models can process vast amounts of biomedical literature, clinical trial data, and genetic information to uncover hidden patterns and generate new insights. In addition to identifying new targets or therapeutics,^166^ LLM-based agentic systems can be used to design and analyze new experiments to develop new therapeutic systems.^167^ In addition to assisting researchers in identifying drug targets, predicting drug interactions, and optimizing therapeutic regimens, LLMs can also be used in clinical settings to support physicians by offering real-time, evidence-based recommendations and improving diagnostic accuracy.

LLMs can also be combined with foundation models to take advantage of their knowledge capabilities. As described earlier, biomedical foundation models have been trained on a broad range of data across multiple therapeutic modalities. In the case of RNA therapeutics, they can be used to incorporate broader knowledge of RNA therapeutics in all three areas (payload, vehicle, and target) to aid in the refinement of more specific aspects of the therapeutic design.^168^

### 7.3 Emerging Trends

While transformer-based approaches have come to dominate large language models, other architectures, such as Hyena-based architectures, have shown utility for biological sequence contexts due to their ability to scale well to long biological sequences.^122^ In addition, recent developments have emphasized hybrid models that combine different neural network types and incorporate multimodal data.^169,170^ These approaches enable the integration of diverse datasets, including gene expression data, chromatin accessibility information, and 3D genome architecture. This integration provides comprehensive insights into biological processes.

Transfer learning has emerged as a valuable approach, where models pre-trained on large genomic datasets are fine-tuned for specific tasks.^171,172^ This method proves particularly effective when working with limited data. Simultaneously, explainable AI approaches are becoming crucial, helping researchers understand model predictions and increasing confidence in deep learning applications.^173^ In addition, semi-supervised learning (a type of learning paradigm that involves providing both labeled and unlabeled data to a model and allowing the model to glean insights from both sets of data) has emerged as a promising solution to data scarcity challenges.^147^ This approach enables more efficient use of limited labeled data, insights from larger unlabeled datasets, and ultimately effective performance in novel applications with sparse labeled data. This is particularly important in biomedical applications such as medical imaging, in which unlabeled data is abundant and labeled data is scarce;^174^ using semi-supervised learning can help extract information from this abundance of unlabeled data to help models make predictions.^175^ The technique has shown promise in RNA therapeutic development,^148,149^ offering a practical approach to model training despite data limitations. New architectures such as the Mamba model^176^ can be employed to improve on existing transformer architectures, with improvements in sequence modeling and faster inference; these can particularly be applied to RNA structure prediction and CRISPR gene editing prediction due to their ability to more efficiently model sequences. New model paradigms such as chain-of-thought reasoning^177^ can improve the ability of transformers and large language models to predict RNA sequence-structure and sequence-function relationships, particularly in out-of-distribution scenarios. Finally, generative models such as diffusion models^178^ and variational autoencoders^179^ can be used to learn how to generate high-performing RNA sequences (particularly in applications such as CRISPR gene editor design and UTR design).

### 7.4 Predictions and Forecasts

In the upcoming decade, deep learning for RNA therapeutics is expected to enable groundbreaking advancements in the design and development of RNA-based drugs. With increasingly accurate models for RNA structure prediction, researchers will have a more comprehensive outlook at their biological functions and interactions. This capability will facilitate the design of more effective siRNAs, ASOs, and mRNA-based vaccines by optimizing their stability, delivery, and target specificity. This could be done by optimizing aspects of therapeutic RNA structure such as the UTRs and RNA modification sites, which can modulate RNA translation and protein production. Deep learning will likely also improve the identification of RBPs and their binding sites, aiding in the development of therapies that modulate RNA-protein interaction. The integration of multi-omics data through deep learning will enhance our ability to understand complex regulatory networks involving RNA, leading to more comprehensive and personalized therapeutic strategies. In addition, high-throughput in vivo screening techniques such as barcoding can provide higher quantities of in vivo efficacy data (especially for RNA delivery methods), which can be used to overcome the generally weak in vitro—in vivo correlation and help train more accurate deep learning models. New methods of tokenization that incorporate the quality of sequencing reads^180^ can be used to convert this data (which can be noisy, particularly in an in vivo setting) into tokens that can be fed into large language models or used to pre-train foundation models with the ability to learn relevant data for RNA therapeutic design.

With regards to model development, we expect that combinations of models can be employed that mitigate some of the weaknesses of existing models, creating hybrid model architectures. For example, generative models (which can produce a large amount of previously unseen RNA structures) can be used in conjunction with other model architectures such as CNNs, RNNs, GNNs, and transformers to train these models using semi-supervised learning;^181^ this could alleviate some concerns with data scarcity. Other models have used combinations of CNNs and RNNs, using the information compression capacity of a CNN to provide relevant data for training an RNN (such as a bidirectional gated recurrent unit) in a more efficient manner.^182^ Finally, combinations of CNNs and transformers can utilize the strengths of each (local interactions for CNNs, long-range interactions for transformers) to more strongly model RNA sequences.^183^

Overall, the integration of deep learning into medical research and practice, as well as its combination with advanced screening techniques for high-quality data generation and model training, will accelerate innovation, enhance the precision of treatments, and ultimately improve patient outcomes.

Figure 4 summarizes some of the potential uses of deep learning in RNA therapeutic design and development in the future.

**Figure 4:**
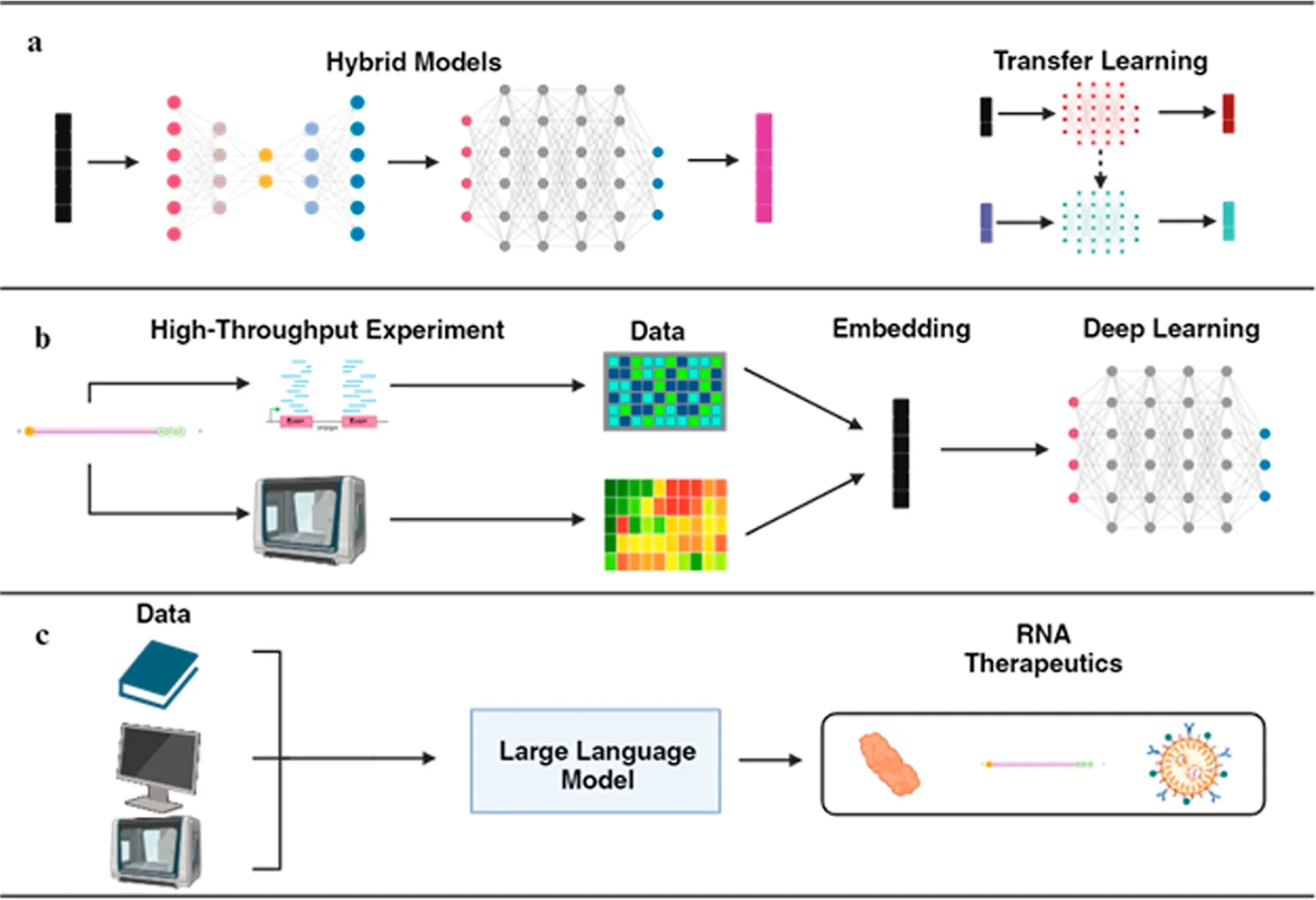
Future perspectives on the use of deep learning for designing and developing RNA therapeutics. In recent years, deep learning for RNA therapeutic design and development has been augmented using new hybrid model architectures and paradigms such as transfer learning (a), large-scale data generation with experimental techniques such as RNA sequencing, high-throughput barcoded assays, and high-throughput LNP transfection assays (b), and large language models that can integrate multimodal data to generate new insights into RNA therapeutics (c). Created in BioRender. Traverso, G. (2024) https://BioRender.com/m15l890.

## 8 Conclusions and Future Directions

The growing importance of RNA therapeutics stems from their demonstrated ability to precisely modulate protein production and treat disease. As more RNA therapies advance through clinical development, optimization efforts will intensify, generating substantial data resources for therapeutic refinement. Deep learning methods are uniquely positioned to leverage this expanding dataset, offering insights into multiple aspects of RNA therapy design and development.

Various deep learning architectures serve distinct yet complementary roles in advancing RNA therapeutics:

- Traditional neural networks identify crucial parameters for therapy design.
- Transformers elucidate both short-range and long-range sequential effects on the RNA structure and gene editing.
- More complex hybrid models combine these architectures to enhance predictive and analytical capabilities.

While deep learning has already demonstrated success in applications such as RNA structure prediction and CRISPR-Cas9 development, several challenges remain:

1. Improving model explainability.
2. Identifying novel therapeutic targets.
3. Expanding into emerging areas like mRNA delivery optimization.

The potential for deep learning to revolutionize RNA therapeutic development is substantial, particularly as new applications emerge and existing approaches are refined. The iterative application of insights across different domains promises to generate increasingly effective solutions for current challenges in RNA therapeutics.

## Notes

From FY 2020 to the present, R.L. received licensing fees (to patents in which he was an inventor on) from, invested in, consulted (or was on Scientific Advisory Boards or Boards of Directors) for, lectured (and received a fee), or conducted sponsored research at MIT for which he was not paid for the following entities: see https://www.dropbox.com/scl/fo/9hrpxuzs72iwvmoqze7rx/AA_OoThWVjAex7X_LhwLRcwrlkey=llza5qcutubtyx34uqrrfy141&st=zcgqc4qp&dl=0. Complete details for G.T. can be found at the following link: https://www.dropbox.com/sh/szi7vnr4a2ajb56/AABs5N5i0q9AfT1IqIJAE-T5a?dl=0.
